# PUM1 in Breast Cancer: Tumor Expression and Prognostic and Predictive Significance

**DOI:** 10.3390/medicina61101810

**Published:** 2025-10-09

**Authors:** Abrar I. Aljohani

**Affiliations:** Department of Clinical Laboratory Sciences, College of Applied Medical Sciences, Taif University, Taif 21944, Saudi Arabia; abrar.g@tu.edu.sa

**Keywords:** breast cancer, prognostic, predictive, PUM1

## Abstract

*Background and Objectives*: Breast cancer (BC) is a complex disease requiring a comprehensive treatment approach due to its diverse characteristics. Critical molecular determinants of BC have been identified using advanced genomic, transcriptomic, and proteomic approaches. Assessing the biomarkers associated with the onset of early-stage BC may help identify the risk of metastasis and inform treatment decisions. A previous bioinformatic analysis using two large BC cohorts identified pumilio RNA binding family member 1 (PUM1) as a key gene in invasive BC. However, no study has yet examined the prognostic and predictive value of PUM1 in invasive BC and its correlation with aggressive tumor behavior. This study aimed to fill this need. *Materials and Methods*: Correlations between *PUM1* expression and patients’ clinicopathological characteristics and outcomes were explored in publicly available BC transcriptomic data acquired using DNA microarrays (*n* = 10,872) and RNA sequencing (*n* = 4421) using BC Gene-Expression Miner v5.0. PUM1 expression in samples from 100 patients with invasive BC at King Abdul Aziz Specialist Hospital, Saudi Arabia, was assessed immunohistochemically. Correlations between PUM1 expression and patients’ clinicopathological characteristics (e.g., age, tumor grade, tumor size, and outcome) were assessed. The online platform ROC Plotter was also used to investigate the predictive significance of PUM1. *Results*: High PUM1 gene and protein expression correlated positively with aggressive features of BC, including high histological grade, high Ki-67 expression, negative hormone receptors, and the triple-negative BC molecular subtype. High PUM1 expression correlated with poor outcomes, and high *PUM1* expression was associated with a lower pathological complete response to anti-endocrine treatment but a high response to chemotherapy. *Conclusions*: These results indicate that PUM1 may serve as a potential prognostic and predictive biomarker in patients with invasive BC. PUM1 may serve as a therapeutic target in BC cases with unfavorable prognoses. However, further validation in larger, multi-center cohorts and further functional assessment are required to deepen our understanding of PUM1’s role in BC.

## 1. Introduction

Breast cancer (BC) continues to be a global public health concern, and its incidence has been increasing over recent decades [1]. Global efforts are needed to mitigate its increasing incidence by identifying biomarkers associated with the development of early-stage BC, which may aid in determining the likelihood of metastasis and guiding treatment options, particularly in transitioning countries, where its incidence is increasing rapidly and mortality rates remain high. Notwithstanding progress in early identification and the development of targeted medicines, many patients encounter treatment failure because of tumor heterogeneity, therapeutic resistance, and the limited effectiveness of current regimens. These issues impose considerable physical, psychological, and financial burdens on patients and healthcare systems [2]. Current research suggests that cancer is both a hereditary and epigenetic disorder. Numerous critical aspects of tumor biology are regulated by epigenetic alterations, influencing the development and dissemination of primary tumors and the immune system’s response within the neoplastic microenvironment [3].

Unlike transcriptional regulation, post-transcriptional gene regulation has not been thoroughly studied in cancer until recently, despite its apparent importance. The Pumilio (PUM) proteins are extensively documented in post-transcriptional gene regulation in various species. They contain the Pumilio-Fem3-binding factor (PUF) RNA-binding domain, which recognizes the UGUANAUA motif generally found in the 3′ untranslated region of target mRNAs. PUM proteins attract protein cofactors, which drive target mRNAs towards translation, splicing, polyadenylation, repression, activation, destruction, or particular localization [4,5]. To modulate the expression of target genes at the post-transcriptional level, RNA binding proteins (RBPs) bind to their mRNAs at specific sites. RBPs are evolutionarily conserved master regulators of mRNA processing and translation that are crucial for translational control, mRNA stability, subcellular localization, post-transcriptional repression, and other processes [6]. Humans have three PUM genes on chromosomes 1 and 2, encoding pumilio RNA binding family members 1 (PUM1), 2 (PUM2) and 3 (PUM3) [7]. Overall, 83% of the amino acids in PUM1 and PUM2 are identical, and 91% in the PUF domain are identical [8]. PUM1 is a sequence-specific RBP that is a member of the eukaryotic PUF family, and it is localized in the cytoplasm [7,9].

Abnormal PUM expression patterns have been observed in various cancers, some of which vary between PUM1 and PUM2 [4,5]. Therefore, it is notable that a new area of research focuses on the role of PUM proteins in specific types of cancer. It has also been shown that PUM proteins regulate the levels of numerous mRNAs encoding proteins frequently disrupted during carcinogenesis, including those involved in the cell cycle, proliferation, and apoptosis [4]. PUM1 was highly expressed and acted as an oncogene by enhancing cell proliferation, migration, and invasion in ovarian cancer [10]. MicroRNA-411-5p functioned as a tumor suppressor by blocking the translation of the *PUM1* mRNA in non-small cell lung cancer, suggesting that PUM1 also acts as an oncogene [11]. A previous bioinformatics investigation of two large transcriptomics datasets for BC revealed that *PUM1* was highly expressed in BC [12]. *PUM1* has also been identified as a potential housekeeping gene in BC [10]. Nonetheless, there is a lack of knowledge about the significance of PUM1 in BC, and its function in controlling carcinogenesis remains inadequately understood. Therefore, it is essential to examine the involvement of PUM1 in BC progression further. Thus, this study aimed to examine *PUM1* expression in BC tissues and its correlation with the aggressive clinical characteristics of BC. It also aimed to evaluate its predictive value and correlation with patient outcomes.

## 2. Materials and Methods

### 2.1. PUM1 Expression in Existing BC Datasets

PUM1 expression was evaluated in publicly annotated BC transcriptomic data acquired using DNA microarrays (*n* = 10,872) and next-generation RNA sequencing (RNA-seq, *n* = 4421) using BC Gene-Expression Miner v5.0 [13]. PUM1 expression and its association with many aggressive characteristics of BC, including molecular subtype, tumor grade, and tumor size, were examined. This tool was also used to evaluate the correlation between PUM1 expression and outcomes via Kaplan–Meier survival analysis.

### 2.2. PUM1 Expression in a New Cohort

PUM1 expression was examined in 100 formalin-fixed paraffin-embedded blocks with sufficient invasive BC tumor tissue from the Histopathology Department at King Abdul Aziz Specialist Hospital (KASH). This study was approved by the Research and Studies Department at KASH for the use of patient tissue (Approval number: 838-02-T-067), and all patients provided informed consent. It also followed the Declaration of Helsinki. The clinicopathological profile of each patient was obtained, including histological grade, tumor size, lymph node status, and age at diagnosis. This cohort also included data on the estrogen receptor (ER), progesterone receptor (PR), human epidermal growth factor 2 (HER2), and marker of proliferation Ki-67 (MKI67). ER/PR status was evaluated using immunohistochemistry (IHC), and tumors were classified as ER^+^/PR^+^ if their staining intensity was >1%. Tumors were classified as HER2^+^ if they scored 3+ on IHC or 2+ on fluorescence in situ hybridization, suggesting *HER2* gene amplification [14]. IHC profiles were used to classify BC molecular subtypes based on St. Gallen subtypes: HER2-enriched (HER2^+^ regardless of ER status), luminal A (ER^+^ and/or PR^+^/HER2^−^, Ki-67 < 20%), luminal B (ER^+^ and/or PR^+^/HER2^−^, Ki-67 ≥ 20%), and triple-negative (ER^−^, PR^−^, and HER2^−^) [14]. Outcome data were collected, including overall survival, which is defined as the time from diagnosis or commencement of therapy to death. Patients in this cohort were treated according to the National Comprehensive Cancer Network guidelines [15].

### 2.3. IHC Staining for PUM1

**Figure 1:**
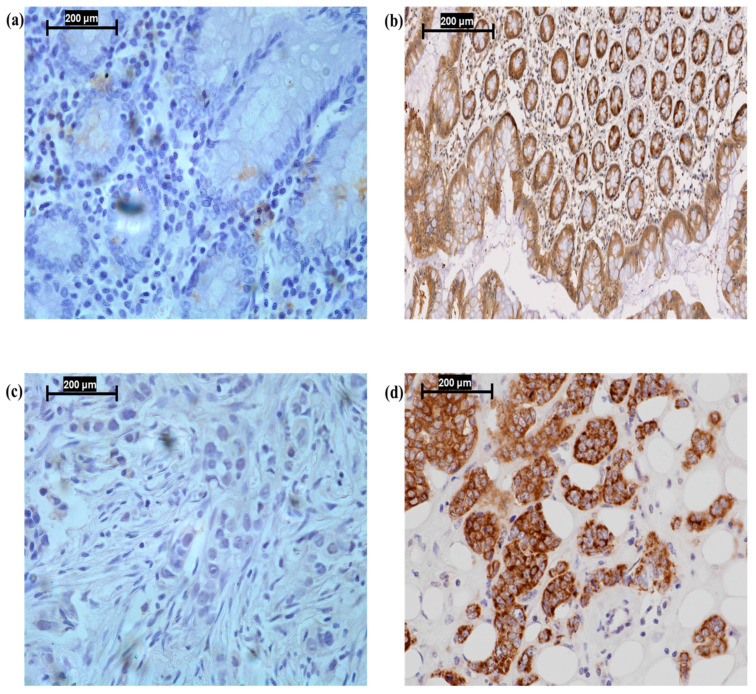
Cytoplasmic expression of PUM1 in invasive BC. (**a**). Negative control (no primary antibody). (**b**). Positive control (colon cancer tissue). (**c**). Negative PUM1 expression. (**d**). Positive PUM1 expression. Magnification: 40×. Scale bars: 200 μm.

For IHC staining of invasive BC tissues, 4-μm slices were obtained using a rotary microtome (Minux^®^ S700; Histo-Line Laboratories, Sugar Land, TX, USA) and placed on positively charged microscope slides. According to the manufacturer, this antibody has been validated by Western blot, which revealed a single band at the predicted molecular weight of PUM1 and no known cross-reactivity. This study validated the specificity by omitting the primary antibody as a negative control, which resulted in no visible staining (Figure 1a). Additionally, the staining of the slides confirmed that the immunoreactivity was mostly confined to the cytoplasm, which is compatible with the known biology of PUM1.

After dewaxing in xylene (X/2050; Fisher Scientific, Loughborough, UK), the sections were rehydrated via an ethanol gradient (E/0665DF; Fisher Scientific, Loughborough, UK) from 100% to 0% (distilled water). Next, the sections were immersed in 100% methanol for 15 min (M/4056; Fisher Scientific, Loughborough, UK) and a 0.9% hydrogen peroxide solution (H/1750; Fisher Scientific, Loughborough, UK) to inhibit endogenous peroxidase activity.

Per the manufacturer’s guidelines for antibody retrieval, microwave energy was used to retrieve the antigens in citrate buffer (pH 6) at 1000 W power for 20 min. Next, the sections were rinsed in phosphate-buffered saline (PBS) and immersed in a blocking solution containing 2% (*w*/*v*) bovine serum albumin (BSA; A4042; Sigma-Aldrich, Haverhill, UK). Then, a 1:50 dilution of the PUM1 primary rabbit polyclonal antibody (A11917; antibodies.com, Cambridge, UK) was applied to the sections in the blocking solution and incubated for 1 h at ambient temperature. After washing the sections with PBS, they were treated with a 1:200 dilution of biotinylated anti-mouse secondary antibody in 2% BSA (PK-6102; Vector Laboratories, Kirtlington, UK) for about 40 min at room temperature. After washing the sections with PBS to eliminate unbound antibodies, they were incubated with the avidin-biotin complex (PK-6100; Vector Laboratories, Newark, CA, USA) for 30 min at ambient temperature. Next, they were incubated with 3,3′-diaminobenzidine (SK-4100; Vector Laboratories, Kirtlington, UK) and then washed with PBS. Then, the slides were rinsed with deionized water and counterstained with Mayer’s hematoxylin (MHS16; Sigma-Aldrich, Glasgow, UK). Next, the slides were rinsed with deionized water and then soaked in various amounts of ethanol, followed by xylene. Finally, the slides were mounted in dibutylphthalate polystyrene xylene (06522; Sigma-Aldrich, Glasgow, UK). The IHC analyses included negative (omission of primary antibody; Figure 1a) and positive (colon cancer tissue; Figure 1b) controls, per the antibody manufacturer’s recommendations.

### 2.4. IHC Assessment of PUM1 Expression

The stained sections were examined under a light microscope at 40× magnification (DMI 3000B; Leica Microsystems, Wetzlar, Germany). Cytoplasmic PUM1 expression was semi-quantitatively evaluated using the modified histochemical (H)-score, where the staining intensity is multiplied by the percentage of positive cells in each tissue section. The H-score ranges from 0 to 300 [16]. Staining intensity was rated as negative (index = 0), weak (index = 1), moderate (index = 2), and strong (index = 3). For each intensity, the proportion of positive cells was assessed subjectively. Non-representative cores were excluded from the scoring, such as those from invasive tumors with <15% core surface area or that were distorted during staining and processing. A professional pathologist worked with the principal researcher to evaluate IHC stained slides in a blinded and independent manner for a minimum of 20% of the assessed cohort. Slides with low inter-rater score concordance were rescored, and the consulting pathologist and principal researcher examined the variations in scores. The level of inter-rater concordance was high for PUM1 and immunoscoring (interclass correlation coefficient [ICC] = 0.90, *p* < 0.001). Due to the non-normal distribution of the PUM1 expression data, the H-score cutoff was established using the median value of 125 to categorize cases into high and low expression groups, a method recognized for its acceptance in biomarker studies and lack of bias.

### 2.5. The Predictive Significance of PUM1 Expression

Whether high PUM1 expression may be inversely correlated with the efficacy of cancer treatments was examined using the online platform ROC Plotter (https://rocplot.org/) (accessed on 15 August 2025). This platform consolidates various transcriptome-level gene expression datasets from the GEO database into a unified database comprising 3104 patients with BC along with response information for diverse treatments, including endocrine therapies, anti-HER2 therapies, and chemotherapeutic agents [17]. The transcriptome data were acquired from patient biopsies before therapy, and patients are categorized into responders and non-responders based on clinical criteria. The responder and non-responder groups were analyzed using two statistical methods: the Mann–Whitney U test (a non-parametric *t*-test) and the receiver operating characteristic (ROC) curve. The ROC curve evaluates the ability of the gene expression model to differentiate between responders and non-responders. It is assessed using the area under the ROC curve (AUC), which indicates the predictive efficacy of the gene. A higher AUC indicates superior model performance in differentiating between responders and non-responders among patients. For cancer biomarkers with prospective clinical use, the AUC must be > 0.6, with an AUC of >0.7 indicative of a high-quality cancer biomarker [17].

### 2.6. Statistical Analysis

The data were statistically analyzed using SPSS Statistics (version 24.0; IBM Corp., Chicago, IL, USA). The degree of concordance between the two raters’ PUM1 scores was assessed using the ICC. Correlations between *PUM1* expression and clinicopathological characteristics were assessed using chi-square tests. Univariate survival analysis was performed using Kaplan–Meier curves and the log-rank test. The Cox regression model was used for multivariate analysis. A two-tailed *p*-value of <0.05 was considered statistically significant.

## 3. Results

### 3.1. PUM1 mRNA Expression and Clinicopathological Parameters

**Figure 2:**
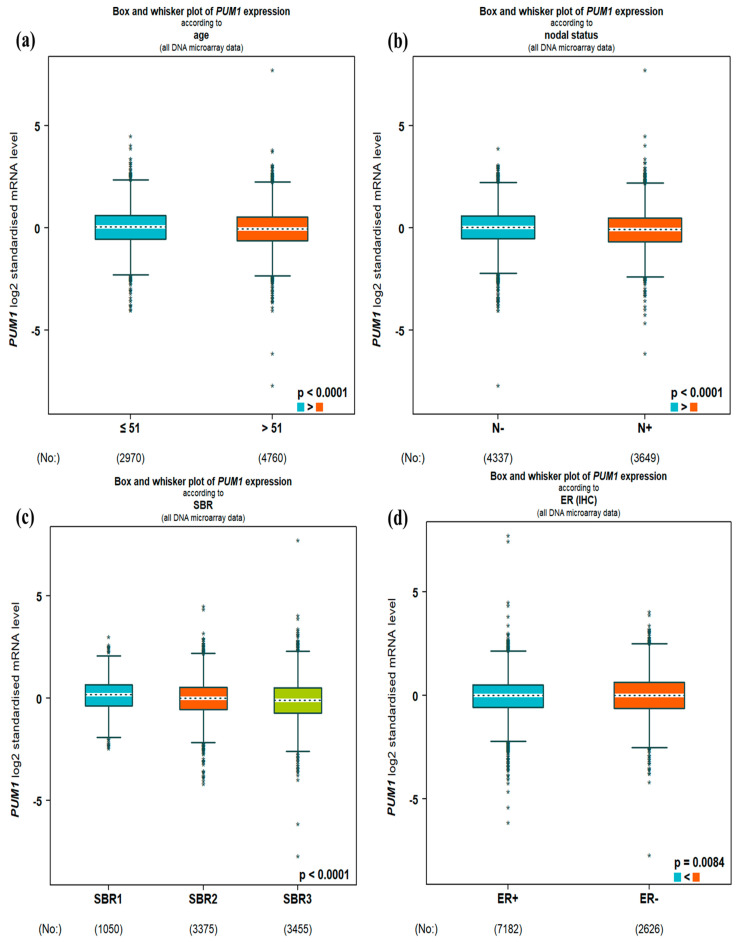

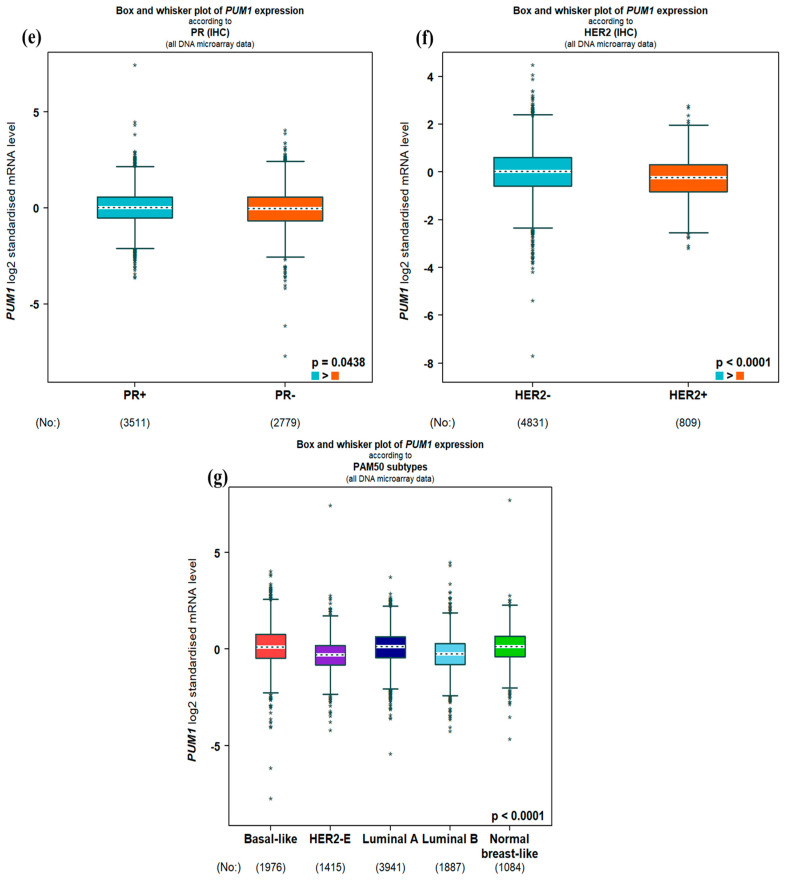
Correlations between PUM1 expression and clinicopathological parameters in the DNA microarray data: (**a**). Patient age: ≤51 (blue) and >51 (red). (**b**). Lymph node status: N^−^ (blue) and N^+^ (red). (**c**). Scarff, Bloom, and Richardson grade: SBR1 (blue), SBR2 (red) and SBR3 (green). (**d**). ER status: ER^+^ (blue) and ER^−^ (red). (**e**). PR status: PR^+^ (blue) and PR^−^ (red). (**f**). HER2 status: HER2^−^ (blue) and HER2^+^ (red). (**g**). PAM50 subtype: Luminal A (dark blue), Luminal B (blue), HER2-enriched (purple), Basal-like (red), and Normal-like (green). * no need to be explained.

**Figure 3:**
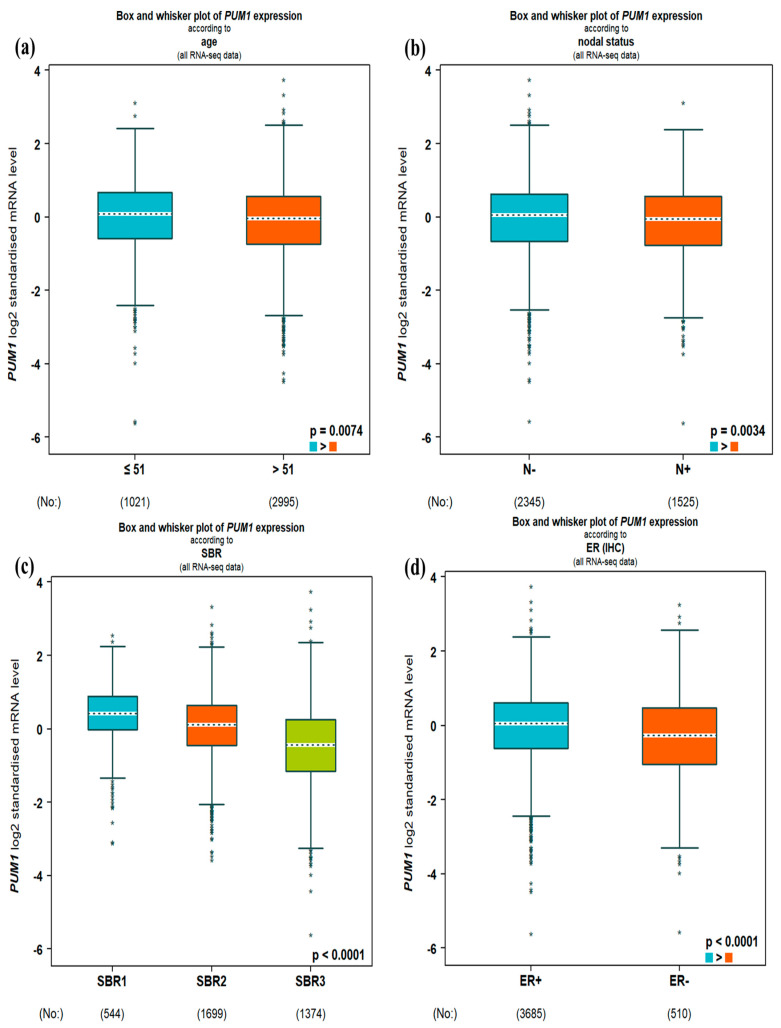

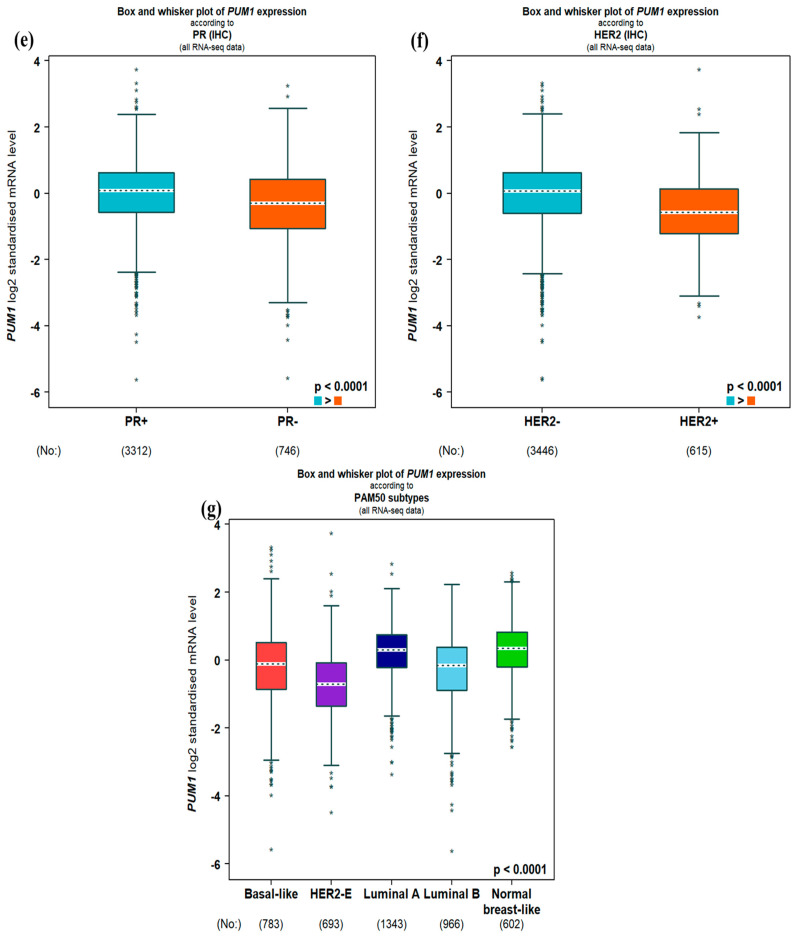
Correlation between PUM1 expression and clinicopathological parameters in the RNA-seq data. (**a**). Patient age: ≤51 (blue) and >51 (red). (**b**). Lymph node status N^−^ (blue) and N^+^ (red). (**c**). Scarff, Bloom, and Richardson grade: SBR1 (blue), SBR2 (red) and SBR3 (green). (**d**). ER status: ER^+^ (blue) and ER^−^ (red). (**e**). PR status: PR^+^ (blue) and PR^−^ (red). (**f**). HER2 status: HER2^−^ (blue) and HER2^+^ (red). (**g**). PAM50 subtype: Luminal A (dark blue), Luminal B (blue), HER2-enriched (purple), Basal-like (red), and Normal-like (green).

In the DNA microarray data, high PUM1 expression was significantly associated with young age, negative lymph nodes, tumor grade 2 (all *p* < 0.0001), ER^−^ (*p* = 0.0084), PR^+^ (*p* = 0.0438), and HER2^−^ (*p* < 0.0001; Figure 2). In the RNA-seq data, high PUM1 expression was significantly associated with young age (*p* = 0.0074), negative lymph nodes (*p* = 0.0034), tumor grade 2 (*p* < 0.0001), ER^+^ (*p* = 0.0084), PR^+^ (*p* < 0.0001), and HER2^−^ (*p* < 0.0001; Figure 3).

Regarding the prediction analysis of microarray 50-gene classifier (PAM50) BC molecular subtypes, in the DNA microarray data, high PUM1 expression correlated strongest with basal-like, followed by luminal A, normal-like, luminal B, and HER2 (all *p* < 0.0001; Figure 2). In the RNA-seq data, high PUM1 expression correlated strongest with luminal A, followed by normal-like, basal-like, luminal B, and HER2 (all *p* < 0.0001; Figure 3).

### 3.2. PUM1 Protein Expression and Clinicopathological Parameters

PUM1 was localized in the cytoplasm of invasive BC tissues, exhibiting no notable membranous or nuclear staining, with intensities ranging from non-existent to high (Figure 1c,d). High PUM1 expression (H-score > 125) was detected in the tissue sections from 50 of the 100 (50%) patients with invasive BC. High PUM1 expression correlated significantly with aggressive features of BC, including tumor grade III (*p* < 0.001), ER^−^ status (*p* = 0.003), PR^−^ status (*p* = 0.002), and Ki-67 positive (*p* = 0.001; Table 1). Regarding the BC molecular subtypes, high PUM1 expression was most strongly associated with triple negative, followed by luminal B, HER2, and luminal A (*p* < 0.001; Table 1).

The Benjamini–Hochberg false discovery rate (FDR) adjustment was conducted; the strongest associations maintained statistical significance (FDR-adjusted *p* < 0.006), but weaker associations lacked significance (FDR-adjusted *p* > 0.1), suggesting exploratory results.

### 3.3. PUM1 Expression and Patient Outcomes

**Figure 4:**
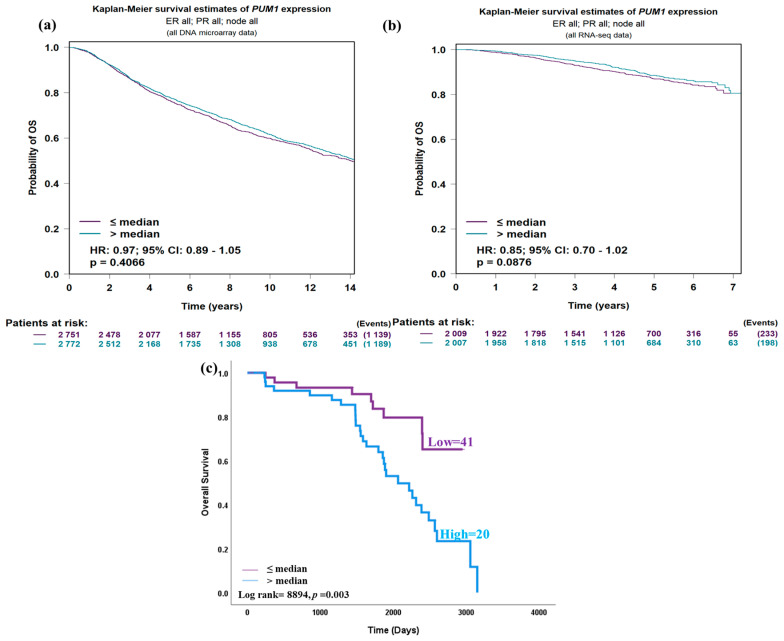
Kaplan–Meier survival analysis of PUM1 expression. (**a**). DNA microarray data (no prognostic significance observed). (**b**). RNA-seq data (no prognostic significance observed). (**c**). KASH cohort (high PUM1 expression associated with worse outcome).

In both the DNA microarray and RNA-seq data, PUM1 gene expression was not associated with overall survival (Figure 4a,b). However, high PUM1 protein expression was associated with worse outcomes in the KASH cohort (p = 0.003; Figure 4c).

In multivariate Cox regression including tumor grade, ER status, PR status, HER2 status, and Ki-67 status as factors, PUM1 protein expression was shown to be an independent prognostic indicator of unfavorable survival regardless of these factors (HR = 3.816, 95% CI = 1.826–10.984, *p* = 0.013; Table 2).

### 3.4. Predictive Value of PUM1 mRNA Expression

**Figure 5:**
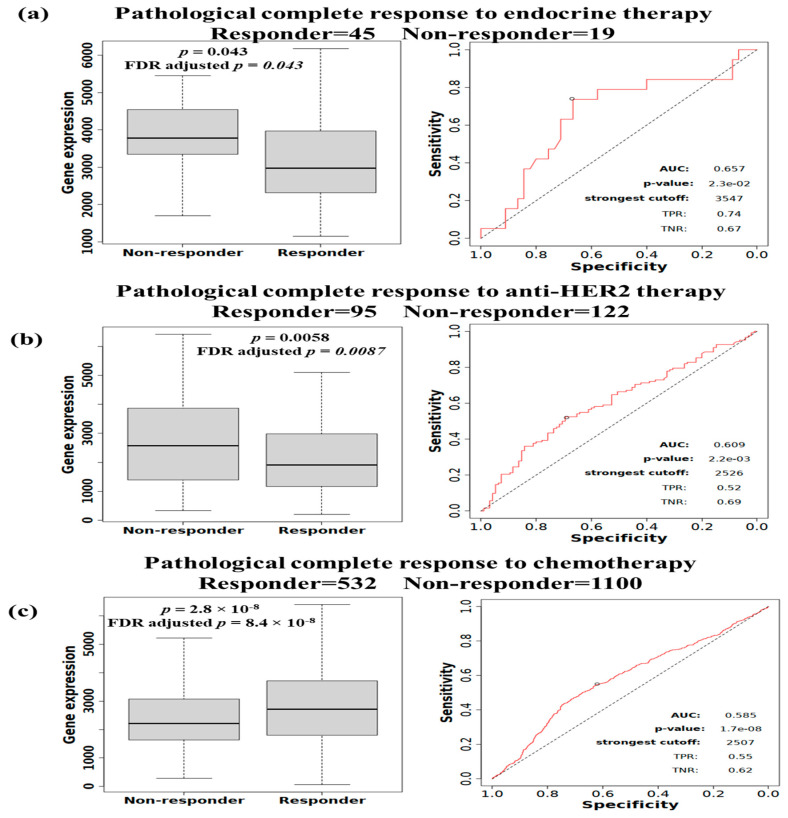
PUM1 expression in patients with BC and their responses to anti-endocrine therapy, anti-HER2 therapy, and chemotherapy. The left panels present box plots of PUM1 expression levels in non-responders and responders. The right panels present ROC plots illustrating the specificity and sensitivity of PUM1 expression as a predictor of the response. (**a**). Anti-endocrine therapy. (**b**). Anti-HER2 therapy. (**c**). Chemotherapy.

Using the ROC Plotter platform, it has been observed that in patients with BC, higher PUM1 expression was associated with a lower pathological complete response to anti-endocrine treatment (Figure 5a; *p* = 0.043, Mann–Whitney U test; AUC = 0.657), an unfavorable response to anti-HER2 treatment (Figure 5b; *p* = 0.0058, Mann–Whitney U test; AUC = 0.609), and a higher pathological complete response to chemotherapy (Figure 5c; *p* = 2.8 × 10^−8^, Mann–Whitney U test; AUC = 0.585). These findings indicate that PUM1 has limited predictive performance.

Following the Benjamini–Hochberg FDR correction for multiple comparisons, the associations of PCR with chemotherapy (FDR-adjusted *p* = 8.4 × 10^−8^), anti-HER2 treatment (FDR-adjusted *p* = 0.0087), and endocrine therapy (FDR-adjusted *p* = 0.043) persisted as statistically significant, demonstrating the robustness of the results.

## 4. Discussion

BC is a complex disease characterized by many molecular subtypes, including hormone receptor-positive, HER2-positive, and triple-negative tumors, each exhibiting distinct responses to systemic therapy. Recent advancements in the comprehension of these subtypes have transformed patient care from a reliance on clinical staging to the integration of genetic profiling, facilitating more accurate prognosis classification and personalized therapy selection [18]. Prognostic or predictive factors can be employed in clinical practice to guide treatment decisions and predict outcomes. Over the years, numerous prognostic markers have been identified in oncology, such as tumor grade, stage, and size [19]. The discovery of prognostic and predictive factors is becoming increasingly relevant in medical research, particularly given the increased understanding of diseases and genetics due to scientific advances, resulting in more targeted therapy [20]. A previous study indicated that PUM1 is among the key genes in BC [12], where PUM1 has been associated with the regulation of carcinogenesis via the PUM1/eIF2 axis by interacting with long non-coding RNAs in breast cancer [21]. However, the prognostic and predictive significance of PUM1 in BC remains unclear. Therefore, this study investigated PUM1 expression in BC tissues and its association with aggressive clinical features of BC. It also analyzed its correlation with patient outcome and its predictive value.

This study revealed that high *PUM1* expression was significantly associated with young age, negative lymph nodes, tumor grade, and the normal and basal-like molecular subtypes. Moreover, high PUM1 expression was significantly associated with the aggressive features of BC, including tumor grade III, negative hormonal receptor status (ER^−^/PR^−^), and the triple-negative molecular subtype. These characteristics are frequently correlated with aggressive tumor biology and poor prognoses. These results agree with previous studies examining other cancers, including pancreatic, colon, and gastric [22,23,24], which showed that *PUM1* expression was higher in tumor than in normal tissues and associated with aggressive features, such as TNM stage.

*Pum1* knockdown inhibited the development of subcutaneous xenograft tumors and reduced the number of metastatic foci and Ki-67-positive cells in the lung tissues of a lung metastasis mouse model. Ki-67 is markedly expressed in malignant cells and serves as a biomarker for cancer growth [22,25]. This study findings are consistent with these observations, demonstrating that high PUM1 expression was significantly associated with high Ki-67 in patients with BC. 

This study further demonstrated the prognostic significance of PUM1 in BC. High PUM1 protein but not gene expression was associated with shorter overall survival. Numerous factors may explain the inconsistency between mRNA and protein levels. Since cellular mRNA levels primarily dictate protein levels, discrepancies may arise between them during prolonged dynamic activities, such as continual growth, reflecting the cell’s steady state [26]. Another factor that may explain this discrepancy is differences in the methods used to quantify the PUM1 expression. While *PUM1* mRNA levels had been quantified using RNA-seq or DNA microarrays, this study assessed PUM1 protein levels in BC tissues using IHC. Additionally, many explanations might account for this disparity. From the perspective of biology, post-transcriptional and post-translational regulatory processes, variations in protein stability, and interactions within the tumor microenvironment may dissociate mRNA abundance from protein levels [27,28]. Methodologically, technical variation in IHC scoring, antibody specificity, and inter-observer discrepancies may affect protein outcomes [29,30]. Furthermore, IHC data in this study originated from a small, single-center cohort in Saudi Arabia, thus limiting generalizability and increasing the likelihood of cohort-specific effects. These considerations emphasize the need for careful interpretation of the results and highlight the relevance of verifying PUM1 expression at both the transcript and protein levels in larger, multi-institutional cohorts. Nonetheless, the prognostic significance of PUM1 found in this study is consistent with findings in other cancers. High PUM1 expression has been associated with poor prognosis in patients with pancreatic cancer [31]. In addition, *PUM1* knockdown in pancreatic cancer cells activated the eukaryotic translation initiation factor 2 alpha kinase 3 (EIF2AK3/PERK)/eukaryotic initiation factor 2 (eIF2)/activating transcription factor 4 (ATF4) signaling pathway, inhibiting cell growth, invasion, and metastasis, and promoting apoptosis [22]. In clinical samples of gastric cancer, high PUM1 expression was also associated with recurrence, metastasis, and poor survival. Moreover, PUM1 causes metabolic reprogramming in gastric cancer by post-transcriptionally regulating the DEP domain-containing mTOR-interacting protein (DEPTOR) [23].

In multivariate Cox regression, the overexpression of PUM1 protein is of independent prognostic significance. This enhances its potential utility as a biomarker in breast cancer.

Substantial efforts are now being made to identify patients who will benefit from chemotherapy. The predictive modelling via ROC analysis revealed modest AUC values (0.585–0.657), suggesting limited predictive performance. However, the results suggest that those with high *PUM1* expression may benefit from chemotherapy if their tumor develops resistance to hormonal therapy. Therefore, based on this evidence, PUM1 could have a potential role in guiding therapy options for patients with BC, which could improve their outcomes.

These data indicate that PUM1 may serve as a potential prognostic and predictive factor in BC and may be pivotal in the metastatic process. Corroborating other studies, the results of this study indicate that PUM1 is associated with aggressive clinicopathological features in invasive BC, potentially facilitating metastatic processes such as proliferation, invasion, and migration. Further research is required to ascertain its viability as a therapeutic target and to examine how it modulates essential downstream biological processes and signaling to control tumor development and progression.

Despite its significant results, this study was limited by the quantity of the samples used in the IHC analysis. While its sample size is adequate, it may affect the generalizability of the findings. Despite some correlations retaining significance post-FDR correction, the limited sample size and numerous comparisons need careful interpretation. This research integrates large public transcriptome datasets with a smaller, single-center IHC cohort. The public databases provide the evaluation of broad patterns and correlations of PUM1 across various populations, whereas the IHC cohort provides preliminary protein-level confirmation within a Saudi Arabian context. Due to the small size and single-center design of the IHC cohort, protein-level results should be regarded as exploratory and hypothesis-generating rather than definitive. Future studies with larger, multi-center populations will be necessary to corroborate these findings and determine their generalizability. Nonetheless, this study is notable as it is among the first to examine the clinicopathological and prognostic implications of PUM1 expression in patients with BC.

## 5. Conclusions

The findings of this study indicate that PUM1 may serve as a potential prognostic biomarker in breast cancer. The ROC analysis indicated limited predictive efficiency; however, multivariate Cox regression revealed that elevated PUM1 expression serves as an independent prognostic factor for poor survival. The results suggest that PUM1 may have therapeutic significance; nevertheless, these findings are preliminary and need confirmation in larger, independent cohorts before PUM1 can be considered a viable clinical tool. Subsequent research should examine whether PUM1 expression might inform therapeutic choices, especially in individuals exhibiting resistance to conventional hormonal therapies. This may result in the formulation of more tailored treatment strategies aimed at enhancing patient outcomes. Subsequent study should investigate the correlations between PUM1, and additional molecular pathways involved in breast cancer progression.

## Figures and Tables

**Table 1:** Associations between PUM1 expression and clinicopathological parameters in the KASH cohort (*n* = 100).

Variable	PUM1 Expression		*χ* ^2^	*p*-Value	FDR Adjusted *p*
	Low (H-Score ≤ 125)	High (H-Score > 125)			
**Age at diagnosis**					
≤50 years >50 years	22 (46.8%) 28 (52.8%)	25 (53.2%) 25 (47.2%)	0.361	0.548	0.645
**Menopausal status**					
Pre-menopausal Post-menopausal	22 (46.8%) 28 (52.8%)	25 (53.2%) 25 (47.2%)	0.361	0.548	0.645
**Tumor size**					
≤10 mm >10 mm	19 (61.3%) 12 (42.9%)	12 (38.7%) 16 (57.1%)	2.005	0.157	0.224
**Tumor grade**					
I II III	8 (88.9%) 38 (67.9%) 1 (3.3%)	1 (11.1%) 18 (32.1%) 29 (96.7%)	38.714	<0.001	0.003
**Lymph node status**					
Negative Positive	15 (60.0%) 9 (34.6%)	10 (40.0%) 17 (65.4%)	3.296	0.069	0.115
**ER status**					
Negative Positive	5 (22.7%) 54 (58.4%)	17 (77.3%) 32 (41.6%)	8.731	0.003	0.006
**PR status**					
Negative Positive	6 (24.0%) 44 (59.5%)	19 (76.0%) 30 (40.5%)	9.400	0.002	0.005
**HER2 status**					
Negative Positive	36 (49.3%) 13 (52.0%)	37 (50.7%) 12 (48.0%)	0.054	0.817	0.817
**Ki-67 status**					
Negative Positive	27 (71.1%) 21 (36.8%)	11 (28.9%) 36 (63.2%)	10.675	0.001	0.003
**IHC subtype**					
ER^+^/HER2^−^ low proliferation ER^+^/HER2^−^ high proliferation Triple-negative HER2^+^	29 (74.4%) 15 (41.7%) 1 (7.1%) 4 (50.0%)	10 (25.6%) 21 (58.3%) 13 (92.9%) 4 (50.0%)	20.534	<0.001	0.003

**Table 2:** Multivariate Cox regression analysis for predictors of overall survival and PUM1 protein expression in the KASH cohort.

Parameters	Hazard Ratio (HR)	95% Confidence Interval (CI)		*p*-Value
		Lower	Upper	
**PUM 1 protein expression**	3.816	1.326	10.984	0.013
**Tumor grade**	0.749	0.374	1.498	0.414
**ER status**	0.753	0.190	2.988	0.687
**PR status**	0.530	0.130	2.154	0.374
**HER2 status**	1.233	0.517	2.941	0.636
**Ki-67 status**	0.677	0.296	1.549	0.355

## Data Availability

Data is unavailable due to privacy or ethical restrictions. Directorate of Health Affairs must grant permission before data can be disclosed.
